# Outcomes of Transcatheter and Surgical Aortic Valve Replacement in Distressed Socioeconomic Communities

**DOI:** 10.7759/cureus.23643

**Published:** 2022-03-30

**Authors:** Michael P Rogers, Anthony J DeSantis, Haroon M Janjua, Sujay Kulshrestha, Paul C Kuo, Lucian Lozonschi

**Affiliations:** 1 Surgery, University of South Florida Morsani College of Medicine, Tampa, USA; 2 Surgery, Loyola University Medical Center, Maywood, USA

**Keywords:** aortic valve surgery, socioeconomic factors, socioeconomic determinants, transcatheter aortic replacement, adult cardiac surgery

## Abstract

Objective

Patients of low socioeconomic status have an increased risk of complications following cardiac surgery. We aimed to identify disparities in patients undergoing aortic valve replacement using the Distressed Communities Index (DCI), a comparative measure of community well-being. The DCI incorporates seven distinct socioeconomic indicators into a single composite score to depict the economic well-being of a community.

Methods

The Healthcare Cost and Utilization Project State Inpatient Database (HCUP-SID) for Florida and Washington was queried to identify patients undergoing surgical and transcatheter aortic valve replacement (surgical aortic valve replacement [SAVR], transcatheter aortic valve replacement [TAVR]) between 2012-2015. Patients undergoing TAVR and SAVR were propensity-matched and stratified based on the quintile of DCI score. A distressed community was defined as those in quintiles 4 and 5 (at-risk and distressed, respectively); a non-distressed community was defined as those in quintiles 1 and 2 (prosperous and comfortable, respectively). Outcomes following aortic valve replacement were compared across groups in distressed communities. Propensity score matching was used to balance baseline covariates between groups.

Results

A total of 27,591 patients underwent aortic valve replacement. After propensity matching, 5,331 patients were identified in each TAVR and SAVR group. Distressed TAVR patients had lower rates of postoperative pneumonia (7.6% vs. 3.8%, p<0.001), sepsis (3.6% vs. 1.9%, p<0.05), and cardiac complications (15.4% vs. 7.5%, p<0.001) when compared to highly distressed SAVR patients. When comparing distressed SAVR and TAVR and low distressed SAVR and TAVR groups, no significant difference was found in postoperative outcomes, except distressed TAVR experienced more cases of UTI.

Conclusions

Highly distressed TAVR patients had lower incidences of postoperative sepsis, pneumonia, and cardiac complications when compared to the highly distressed SAVR cohort. Patients undergoing TAVR in highly distressed communities had an increased incidence of postoperative urinary tract infection. DCI may be a useful adjunct to current risk scoring systems.

## Introduction

This article was presented in poster format at the International Society for Minimally Invasive Cardiothoracic Surgery Annual Meeting in June 2021.

Low socioeconomic status is associated with an increased risk of all-cause mortality, cardiovascular morbidity, and mortality, as well as complications following cardiac surgical procedures [1-4]. Determinants including educational level, employment status, housing, and yearly income are commonly reported when evaluating the impacts of socioeconomic status on health outcomes. While these factors may provide some aggregate context, the complete collection of influences is often incomplete. As a consequence, the appropriate evaluation and integration of these data into outcomes research is imperfect. To this end, the Economic Innovation Group has developed the Distressed Communities Index (DCI) to better understand and incorporate these factors. The DCI combines seven distinct and complementary socioeconomic indicators into a single score to depict the economic well-being of a community [5]. These scores range from 0 (no distress) to 100 (severe distress) and account for employment, housing vacancy, poverty rate, educational level, local job growth, business development, and median household income [5].

Recent analysis has demonstrated an increased risk of adverse events and death in patients from distressed communities following coronary artery bypass grafting (CABG) [2]. We sought to evaluate distressed patients undergoing surgical and transcatheter aortic valve replacement to better elucidate potential disparities in this cohort. Further, we sought to compare outcomes of surgical versus transcatheter aortic valve replacement (SAVR, TAVR) in patients with high socioeconomic distress as quantified by the Distressed Communities Index. We propose two hypotheses for investigation: first, patients from communities with higher socioeconomic distress will experience worse outcomes following aortic valve replacement as compared to patients from less distressed communities. Second, despite this proposed relationship between increased distress and worse outcome, the lower surgical morbidity and shorter length of stay associated with TAVR will serve a protective effect as compared to SAVR in regard to the outcomes of patients from the most socioeconomically distressed communities.

## Materials and methods

Data source and study population

The Healthcare Cost and Utilization Project State Inpatient Database (HCUP-SID) for Florida and Washington was queried to identify patients undergoing surgical and transcatheter aortic valve replacement between 2012 and 2015. HCUP-SID is an administrative dataset maintained by the Agency for Healthcare Research and Quality that provides longitudinal data on inpatient encounters for healthcare facilities in the respective state [6]. Patients were queried based on the International Classification of Diseases, Ninth Revision, Clinical Modification Volume 3 (ICD-9-CM3) and International Classification of Diseases, Tenth Revision, Procedure Coding System (ICD-10-PCS) procedure codes for surgical aortic valve replacement (ICD-9-CM 35.21 and 35.22 and ICD-10-PCS codes 02RD07Z, 02RF08Z, 02RF0KZ, 02RF47Z, 02RF48Z, 02RF4KZ, X2RF032, X2RF432) and transcatheter aortic valve replacement (ICD-9-CM 35.05 and 35.06 and ICD-10-PCS codes 02RD37H, 02RF37Z, 02RF38H, 02RF3JH, 02RF3KH, 02RF3KZ) in the HCUP-SID database. Queried International Classification of Disease codes without corresponding patients in HCUP-SID were eliminated from the analysis. Patient records were then linked to the Distressed Communities Index.

DCI covers nearly 25,500 zip codes and 99% of the United States population and is composed of 7 metrics to form a single summary statistic: adults not working, poverty rate, housing vacancy rate, median household income, change in employment, change in establishments, and no high school diploma [5]. These parameters are built from the American Communities Survey 2014 five-year estimates and the Census Bureau County and Zip Code Business Patterns. Each community’s seven rankings are averaged and weighted to create a preliminary score which is then normalized to a final score from 0 (most prosperous) to 100 (most distressed) [5]. Communities were then sorted into five quintiles of economic well-being: prosperous, comfortable, mid-tier, at risk, and distressed (quintile 1 through quintile 5) [5]. A highly distressed community was defined as those in quintiles 4 and 5, a low distressed community was defined as those in quintiles 1 and 2.

Statistical analysis

Continuous variables are reported as mean and standard deviation or median and interquartile range (IQR) and were compared using Student’s t-test for pairwise comparisons. Categorical variables are reported as total and percentage (n, %) and compared using the chi-square test when appropriate.

Inverse probability of treatment weighting (IPTW) propensity score matching was utilized to balance baseline covariates between SAVR and TAVR cases [7]. Patients undergoing SAVR and TAVR were propensity-matched 1:1 based on patient age, sex, Charlson Comorbidity Index (CCI), and preexisting conditions, including acute myocardial infarction, congestive heart failure, peripheral vascular disease, diabetes with and without complications, cerebrovascular disease, and chronic obstructive pulmonary disease. The Stata CCI module was used to calculate the CCI from data containing ICD-9-CM diagnoses codes, which groups the comorbidity score into low, moderate, and severe (CCI-0, CCI-1, CCI-2). Highly distressed communities (defined as patients in quintiles 4 and 5) were evaluated between SAVR and TAVR groups to determine differences in hospital length of stay, number of pre-operative chronic conditions, and post-operative outcomes. To determine the impact of highly distressed communities, highly distressed cohorts were compared to low distressed patients (quintiles 1 and 2) within SAVR and TAVR groups separately, by 1:1 inverse probability of treatment weighting propensity matching based on the previously aforementioned factors.

All data preparation, merging, and cleaning was performed using SPSS Inc. Released 2007. SPSS for Windows, Version 16.0. Chicago, SPSS Inc. All modeling, including propensity matching, was performed in R Studio Statistical Analysis software; software included the following four packages: readstata13, tableone, MatchIt, and Matching. The “Matchit” package in R statistical software was used to perform propensity matching, which improves robustness and decreases the dependence of causal inferences on statistical modeling assumptions.

## Results

A total of 27,591 patients were identified as having undergone aortic valve replacement with either open surgical or transcatheter technique from 95 hospitals in Florida and Washington between 2012 and 2015. After propensity matching, 10,662 total patients were included for analysis. Patients were then stratified by Distressed Communities Index score; 1,347 SAVR and 1,355 TAVR patients were identified in quintiles 4 and 5 (highly distressed), and 2,763 SAVR and 2,794 TAVR patients in quintiles 1 and 2 (low distressed). Overall, TAVR patients were older (81.5 vs. 79.6 years), had a shorter length of stay (7.7 vs. 11.9 days), and had a higher mean number of pre-operative chronic comorbidities (9.7 vs. 10.1 co-morbid conditions) (Table 1). Patients undergoing surgical aortic valve replacement had a higher proportion of patients having private health insurance as a primary payer, male sex, Hispanic or Black ethnicity, and had more hospital admissions classified as “emergent” as compared to the overall transcatheter aortic valve replacement group.

**Table 1:** Patient characteristics of overall aortic valve replacement cohort for surgical and transcatheter aortic valve replacement

Patient Characteristics	SAVR n = 22,260	TAVR n = 5,331	p-value^†^
Age	69.6 (12.5)	81.6 (8.7)	<0.001
Number of Chronic Conditions	8.3 (3.2)	10.1 (3.3)	<0.001
Length of Stay	10.1 (8.6)	7.8 (7.05)	<0.001
Distressed Communities Index Score	41.6 (25.1)	41.2 (25.3)	0.283
Patient Sex			<0.001
Male	14,376 (64.6)	2,949 (55.3)	
Female	7,884 (35.4)	2,382 (44.7)	
Race			<0.001
White	18,761 (84.3)	4,697 (88.1)	
Black	876 (3.9)	126 (2.4)	
Hispanic	2,061 (9.3)	354 (6.7)	
Others	562 (2.5)	154 (2.9)	
Primary Payer			<0.001
Medicare	15,743 (70.7)	4,934 (92.6)	
Medicaid	981 (4.41)	42 (0.79)	
Private Insurance	4,647 (20.9)	277 (5.2)	
Others	889 (3.99)	78 (1.5)	
Admission Type			<0.001
Emergency	3,392 (15.2)	402 (7.5)	
Urgent	3,056 (13.7)	722 (13.5)	
Elective	15,812 (71.03)	4,207 (78.9)	
Charlson Comorbidity Index			<0.001
Low Sickness	5,944 (26.7)	553 (10.37)	
Moderate Sickness	6,191 (27.8)	904 (16.96)	
Severe Sickness	10,125 (45.5)	3,874 (72.7)	
Patient Location			0.001
Large metropolitan with at least 1 million residents	11,636 (52.3)	2,907 (54.5)	
Small metropolitan with less than 1 million residents	8,402 (37.7)	1,867 (35)	
Micropolitan areas	1,605(7.2)	379 (7.1)	
Not metropolitan or micropolitan	617(2.8)	178 (3.3)	
Median Household Income State Quartiles			0.159
Quartile 1 (Poorest)	5,329 (23.9)	1,277 (23.95)	
Quartile 2	5,905 (26.53)	1,379 (25.9)	
Quartile 3	6,051 (27.18)	1,410 (26.5)	
Quartile 4 (Wealthiest)	4,975 (22.35)	1,265 (23.7)	
Patient Disposition at Discharge			<0.001
Routine	5,812 (26.1)	1,689 (31.7)	
Transfer to Short-term Hospital	187 (0.8)	35 (0.7)	
All Other Facilities: Skilled Nursing, Intermediate Care	6,871 (30.9)	1,698 (31.9)	
Home Health Care	8,602 (38.6)	1,708 (32.04)	
Against Medical Advice	32 (0.14)	<11	
Deceased	756 (3.4)	199 (3.73)	
Comorbidities			
Acute Myocardial Infarction	2,320 (10.42)	767 (14.39)	<0.001
Congestive Heart Failure	7,178 (32.3)	3,592 (67.4)	<0.001
Peripheral Vascular Disease	3,608 (16.2)	1,143 (21.4)	<0.001
Diabetes Without Complications	5,712 (25.7)	1,554 (29.2)	<0.001
Diabetes With Complications	772 (3.5)	268 (5.03)	<0.001
Cerebrovascular Disease	2,132 (9.6)	726 (13.6)	<0.001
Chronic Obstructive Pulmonary Disease	4,852 (21.8)	1,979 (37.12)	<0.001
Statistics are presented as: mean (standard deviation), n (%)			
^†^Statistical tests performed: Student’s T-Test, Chi-square test of independence			
In compliance with the Healthcare Utilization Project Data Use Agreement (HCUP DUA), cells with fewer than 10 observations are marked “<11”			

Propensity matched overall TAVR, and SAVR patients revealed similar results with respect to age (81.6 vs. 79.5 years, p < 0.001), number of chronic conditions (10.1 vs. 9.7, p < 0.001), and length of hospital stay (7.8 vs. 12 days, p < 0.001). The mean DCI score for the total propensity-matched population was 41.37 ± 25.03 (Table 2). Propensity matched TAVR patients had lower rates of post-operative sepsis, pneumonia, deep vein thrombosis, surgical site infections, and cardiac complications (p < 0.05).

**Table 2:** Distribution of SAVR and TAVR patients with respect to distressed communities index quintiles (unmatched and propensity matched data)

Unmatched Data			
Distressed Quintiles	SAVR n (%)	TAVR n (%)	Total n (%)
1	5,326 (23.9)	1,278 (23.97)	6,604 (23.9)
2	6,179 (27.8)	1,516 (28.4)	7,695 (27.9)
3	4,989 (22.4)	1,182 (22.2)	6,171 (22.4)
4	3,761 (16.9)	874 (16.4)	4,635 (16.8)
5	2,005 (9.01)	481 (9.02)	2,486 (9.01)

1:1 Propensity Matched Data			
Distressed Quintiles	SAVR n (%)	TAVR n (%)	Total n (%)
1	1,200 (22.5)	1,278 (23.97)	2,478 (23.2)
2	1,563 (29.3)	1,516 (28.4)	3,079 (28.9)
3	1,221 (22.9)	1,182 (22.2)	2,403 (22.5)
4	883 (16.6)	874 (16.4)	1,757 (16.5)
5	464 (8.7)	481 (9.02)	945 (8.9)

Highly distressed communities' outcomes

Patient outcomes between highly distressed TAVR and SAVR groups were evaluated based on the highly distressed communities index score, defined as quintiles 4 and 5 (DCI score ≥ 60). Highly distressed patients in the TAVR cohort were older (80.97 vs. 78.6 years, p < 0.001), had a shorter length of stay (8.2 vs. 12.1 days, p < 0.001), had more pre-operative chronic conditions (10.3 vs. 9.8 chronic conditions, p < 0.001), and had a higher, though not statistically significant, the proportion of patients with a high Charlson Comorbidity Index (75.9% vs. 72.9%, p > 0.05) as compared to the highly distressed SAVR cohort. There were no differences with respect to postoperative mortality, pulmonary embolism, myocardial infarction, urinary tract infections, deep vein thrombosis, or surgical site infections. The highly distressed SAVR cohort had increased rates of postoperative pneumonia (7.6% vs. 3.8%, p < 0.001), sepsis (3.6% vs. 1.9%, p < 0.05), and cardiac complications (15.4% vs. 7.5%, p < 0.001) compared to the highly distressed TAVR cohort, though TAVR patients experienced higher rates of procedural complications (1.9% vs. 0.5%, p < 0.05) (Table 3).

**Table 3:** Comparison of SAVR and TAVR propensity matched data for Distressed Communities Index quintiles 4 and 5

Patient Characteristics	SAVR Q4Q5 n = 1,347	TAVR Q4Q5 n = 1,355	p-value^†^
Age	78.58 (8.19)	80.97 (8.52)	<0.001
Number of Chronic Conditions	9.8 (3.10)	10.30 (3.34)	<0.001
Length of Stay	12.2 (8.99)	8.2 (7.35)	<0.001
Distressed Communities Index Score	75.7 (10.01)	76.4 (10.35)	0.09
Mortality			0.466
Deaths	67 (4.97)	64 (4.72)	0.762
Charlson Comorbidity Index			0.224
Low Sickness	138 (10.2)	122 (9.00)	
Moderate Sickness	226 (16.8)	205 (15.1)	
Severe Sickness	983 (72.98)	1028 (75.9)	
Post-Operative Complications			
Pulmonary Embolism	<11	<11	0.177
Myocardial Infarction	<11	<11	0.991
Sepsis	49 (3.6)	26 (1.9)	0.007
Urinary Tract Infection	62 (4.6)	78 (5.8)	0.176
Pneumonia	103 (7.7)	52 (3.8)	<0.001
Deep Vein Thrombosis	<11	<11	0.161
Surgical Site Infection	11 (0.8)	<11	0.129
Cardiac Complications	208 (15.4)	101 (7.5)	<0.001
Procedural Complications	<11	26 (1.9)	<0.001
Statistics are presented as: mean (standard deviation), n (%)			
^†^Statistical tests performed: Students T-Test, Chi-square test of independence			
In compliance with the Healthcare Utilization Project Data Use Agreement (HCUP DUA), cells with fewer than 10 observations are marked “<11”			

Comparisons between high and low distressed communities

Low distressed communities (quintiles 1 and 2) were compared to highly distressed communities (quintiles 4 and 5) within TAVR and SAVR groups separately. Low distressed communities undergoing surgical aortic valve replacement had shorter lengths of hospital stay (9.97 vs. 10.61 days, p < 0.001) but did not differ from the highly distressed SAVR cohort with several pre-operative chronic conditions, mortality, or postoperative complications (Table 4).

**Table 4:** Comparison of SAVR low distressed (Q1Q2) versus high distressed (Q4Q5) propensity matched data

Patient Characteristics	SAVR Q1Q2 n = 5,766	SAVR Q4Q5 n = 5,766	p-value^†^
Age	69 (12.8)	68.7 (12.7)	0.239
Number of Chronic Conditions	8.46 (3.2)	8.4 (3.2)	0.69
Length of Stay	9.97 (8.3)	10.6 (9.03)	<0.001
Distressed Communities Index Score	21.01 (10.8)	75.8 (10.1)	<0.001
Mortality			
Deaths	184 (3.2)	199 (3.5)	0.436
Charlson Comorbidity Index			0.958
Low Sickness	1,411 (24.5)	1,402 (24.3)	
Moderate Sickness	1,557 (27)	1,570 (27.2)	
Severe Sickness	2,798 (48.5)	2,794 (48.5)	
Post-Operative Complications			
Pulmonary Embolism	15 (0.3)	13 (0.23)	0.705
Myocardial Infarction	20 (0.4)	25 (0.4)	0.455
Sepsis	159 (2.8)	181 (3.1)	0.226
Urinary Tract Infection	149 (2.6)	163 (2.8)	0.422
Pneumonia	303 (5.3)	333 (5.8)	0.221
Deep Vein Thrombosis	48 (0.8)	37 (0.6)	0.231
Surgical Site Infection	29 (0.5)	37 (0.6)	0.323
Cardiac Complications	717 (12.4)	712 (12.4)	.888
Procedural Complications	54 (0.9)	54 (0.9)	1
Statistics are presented as: mean (standard deviation), n (%)			
^†^Statistical tests performed: Students T-Test, Chi-square test of independence			
In compliance with the Healthcare Utilization Project Data Use Agreement (HCUP DUA), cells with fewer than 10 observations are marked “<11”			

Low distressed communities did not differ significantly from highly distressed communities in the TAVR cohort with respect to age, several pre-operative chronic conditions, length of hospital stay, mortality, or most post-operative outcomes. However, patients from highly distressed communities had higher rates of postoperative urinary tract infection (5.76% vs. 4.06%, p < 0.05) (Table 5).

**Table 5:** Comparison of TAVR low distressed (Q1Q2) versus high distressed (Q4Q5) propensity matched data

Patient Characteristics	TAVR Q1Q2 n = 1,355	TAVR Q4Q5 n = 1,355	p-value^†^
Age	80.60 (9.5)	80.97 (8.5)	0.289
Number of Chronic Conditions	10.2 (3.4)	10.3 (3.3)	0.235
Length of Stay	7.7 (6.7)	8.2 (7.4)	0.10
Distressed Communities Index Score	20.7 (10.7)	76.4 (10.4)	<0.001
Mortality			
Deaths	53 (3.9)	64 (4.7)	0.299
Charlson Comorbidity Index			0.366
Low Sickness	143 (10.6)	122 (9)	
Moderate Sickness	209 (15.4)	205 (15.1)	
Severe Sickness	1,003 (74.02)	1,028 (75.9)	
Post-Operative Complications			
Pulmonary Embolism	<11	<11	0.563
Myocardial Infraction	10 (0.74)	<11	0.636
Sepsis	20 (1.5)	26 (1.92)	0.372
Urinary Tract Infection	55 (4.06)	78 (5.8)	0.041
Pneumonia	48 (3.5)	52 (3.8)	0.684
Deep Vein Thrombosis	<11	<11	0.365
Surgical Site Infection	<11	<11	1
Cardiac Complications	118 (8.7)	101 (7.5)	0.231
Procedural Complications	24 (1.8)	26 (1.9)	0.775
Statistics are presented as: mean (standard deviation), n (%)			
^†^Statistical tests performed: Students T-Test, Chi-square test of independence			
In compliance with the HCUP DUA, cells with fewer than 10 observations are marked “<11”			

## Discussion

Low socioeconomic status is negatively associated with overall health outcomes in every age category and has been shown to consistently affect communities across developed countries [8,9]. We aimed to evaluate the impact of low socioeconomic status on aortic valve replacement outcomes using a composite measure of socioeconomic distress in a state-wide database analysis of Florida and Washington. Overall, TAVR patients had fewer episodes of postoperative sepsis, pneumonia, and cardiac complications as compared to the SAVR cohort, including when comparing highly distressed (DCI score ≥ 60) communities. Patients undergoing TAVR in highly distressed communities had an increased incidence of postoperative urinary tract infection when compared to a lower distressed population.

Several factors contribute to overall patient outcomes, including individual, nursing, physician, hospital, infrastructure, information technology, and environment, among others. DCI scoring seeks to better elucidate community and environmental influences in a quantifiable way to identify and rectify disparities. Highly distressed communities may have less access to routine medical care or surgical follow-up by nature of their proximity to medical centers and primary care facilities. Previous efforts have highlighted these disparities and identified worse outcomes in patients undergoing coronary artery bypass grafting, left ventricular assist device implantation, and open abdominal aortic aneurysm repair in distressed, rural, and lower socioeconomic status communities [2,10-12]. This analysis adds to the growing body of literature identifying disparate outcomes between these groups.

It is increasingly becoming recognized that healthcare is not delivered in a vacuum and that beyond the individual attributes of the patient, the medical team, hospital, and the delivery and effectiveness of medical care are strongly influenced by the effect of the larger healthcare macroenvironment in which it is delivered. For every patient presenting from a relatively prosperous socioeconomic community, there will be one and if not many presenting from more disadvantaged and distressed communities. While previous analyses have evaluated the potential benefit of minimally invasive techniques on morbidity and mortality on an individual level, this research suggests that minimally invasive techniques such as TAVR may provide a relatively greater benefit in patients who suffer from greater socioeconomic needs. Although the precise mechanism by which distressed communities affect individual patient outcomes remains somewhat elusive, consideration of a patient’s socioeconomic status should be kept in mind in the pre-operative evaluation of patients presenting for aortic valve replacement. Those patients in communities of greater socioeconomic distress may benefit from interventions with relatively limited invasiveness and potential for morbidity.

The Society of Thoracic Surgeons (STS) Risk Calculator and the American College of Cardiology/Society of Thoracic Surgeons Transcatheter Valve Therapy TAVR In-Hospital Mortality Risk Calculator remain the gold standard risk scoring calculators available for evaluating patient risk for surgical and transcatheter aortic valve replacement, respectively [13,14]. While robust, these scoring systems lack the granularity of incorporating in-depth socioeconomic factors in predicting patient outcomes. DCI, or similar surrogates of socioeconomic status, may be useful additions to these current scoring approaches and may strengthen their ability to predict morbidity and mortality. Charles and colleagues’ analysis of DCI as a predictor of risk-adjusted mortality following CABG and Mehaffey and colleagues’ efforts to utilize DCI in scoring systems in the bariatric, cardiac, vascular, and general surgical populations are important highlights toward this goal [15-17].

Several limitations of our analysis should be considered. Notably, this is a retrospective administrative database analysis with inherent limitations thereof. Analysis was confined to the available variables for the study period in the HCUP-SID dataset for Florida and Washington and is therefore subject to selection bias. Additionally, the study period was during the relatively new introduction of TAVR technology and available to most high or moderate risk surgical patients as defined by the Society of Thoracic Surgeons risk scoring schema [18,19]. Accordingly, patients undergoing TAVR for the study period were generally frailer, of advanced age, and had additional co-morbidities. Differences in outcomes between both cohorts may have been influenced by differences in treatment strategy, thus diminishing the role of socioeconomic status on the outcomes measured. Additionally, the Distressed Communities Index, while an exemplary model of community-level factors, cannot account for all community-related influences, and thus certain relevant features may not be accounted for in our analysis.

While acknowledging these limitations, we feel that this study is unique and well-constructed in several ways. The inclusion of patients from 95 hospitals across two distinct and geographically distributed states, Florida and Washington, helped to increase the generalizability of our findings on a broader level. Both Florida and Washington possess diverse patient populations residing in several unique municipalities spanning a wide spectrum of socioeconomic resources (or lack thereof). Furthermore, the inclusion of propensity matching before analysis allows for a more discrete investigation of the role of socioeconomic distress on aortic valve replacement while mitigating confounding variables as much as able.

## Conclusions

Socioeconomic distress is a contributing factor in patient care outcomes. Patients from highly distressed communities may have worse outcomes when compared between surgical and transcatheter aortic valve replacement groups and have an increased incidence of urinary tract infection when compared between low and highly distressed TAVR groups. DCI, a composite score consisting of seven distinct socioeconomic contributors, may be an important addition to existing surgical risk scores for surgical and transcatheter aortic valve replacement to better predict postoperative outcomes.

## Appendices

Appendix 1

Supplementary Material

R Studio Statistical Analysis programming coding for this project is available for review at the following URL: https://github.com/onetomapanalytics/SAVR_TAVR_DCI
